# Ribosomal Alteration-Derived Signals for Cytokine Induction in Mucosal and Systemic Inflammation: Noncanonical Pathways by Ribosomal Inactivation

**DOI:** 10.1155/2014/708193

**Published:** 2014-01-02

**Authors:** Yuseok Moon

**Affiliations:** ^1^Laboratory of Mucosal Exposome and Biomodulation, Department of Microbiology and Immunology, Pusan National University School of Medicine, Yangsan 626-870, Republic of Korea; ^2^Immunoregulatory Therapeutics Group in Brain Busan 21 Project, Busan 609-735, Republic of Korea; ^3^Medical Research Institute, Pusan National University, Busan 609-735, Republic of Korea

## Abstract

Ribosomal inactivation damages 28S ribosomal RNA by interfering with its functioning during gene translation, leading to stress responses linked to a variety of inflammatory disease processes. Although the primary effect of ribosomal inactivation in cells is the functional inhibition of global protein synthesis, early responsive gene products including proinflammatory cytokines are exclusively induced by toxic stress in highly dividing tissues such as lymphoid tissue and epithelia. In the present study, ribosomal inactivation-related modulation of cytokine production was reviewed in leukocyte and epithelial pathogenesis models to characterize mechanistic evidence of ribosome-derived cytokine induction and its implications for potent therapeutic targets of mucosal and systemic inflammatory illness, particularly those triggered by organellar dysfunctions.

## 1. Introduction

As the functional organelle for protein synthesis, ribosomes bound to the endoplasmic reticulum (ER) perform complex surveillance of various pathologic stresses [1–3]. Ribosomal alteration by endogenous and external insults can trigger a variety of pathogenic processes, including inflammatory responses [4–6]. Ribosomal inactivation can be induced by a large family of ribonucleolytic proteins that cleave 28s ribosomal RNA at single phosphodiester bonds within a universally conserved sequence known as the sarcin-ricin loop, which leads to the dysfunction of peptidyltransferase and subsequent global translational arrest [7, 8]. These ribosome-inactivating proteins (RIPs) are enzymes isolated mostly from plants and some of RIPs such as ricins and shiga toxins are potent cytotoxic biological weapons causing tissue injuries and inflammatory diseases [9, 10]. Similar ribosomal RNA injuries have been observed during nonprotein ribosome-inactivating stress triggered by physical and chemical insults such as ultraviolet (UV) irradiation, trichothecene mycotoxins (mostly cereal contaminants produced by molds such *Fusarium* species), palytoxin (an intense vasoconstrictor produced by marine species including dinoflagellate *Ostreopsis ovata*), and anisomycin (an antibiotic produced by *Streptomyces griseolus*), which also interfere with peptidyltransferase activity by directly or indirectly modifying 28s rRNA [11, 12]. The primary action of most ribosome-inactivating stress is the functional inhibition of global protein synthesis; therefore, highly dividing tissues such as lymphoid tissue and mucosal epithelium are the most susceptible targets of the stress [13–15]. Although acute high levels of toxic insults lead to sepsis-like symptoms including hemolytic uremic syndrome [16, 17], several epidemiological studies have suggested that there are also links between ribosome-inactivating stress and human mucosal epithelial illnesses ranging from acute mucosal inflammatory disease to chronic illness, including epithelial malignancy [18–20]. Ribosome-inactivating stress has been investigated in various experimental models as an etiological factor of inflammatory diseases such as ulcerative colitis and hemolytic uremic syndrome [17, 21, 22]. Moreover, upper airway inflammation such as intranasal neutrophilic rhinitis, which is characterized by mucus hypersecretion, atrophy, and exfoliation of transitional epithelium, is also triggered by some of ribosome-inactivating trichothecenes [23–26]. Among a variety of mediators of the ribosomal inactivation-associated pathogenesis, proinflammatory cytokines play key roles in both mucosal and systemic inflammatory responses to ribosome-inactivating stress [27–30]. Although the primary outcome of the insulted cells is the global protein synthesis inhibition due to the ribosomal RNA cleavage and modification [31], the insult leads to some exceptional production of proteins such as cytokines important for cellular homeostasis as well as a variety of pathogenic processes involved in cell survival modulation, proliferation, and stress response [32, 33]. The present review described the mechanistic patterns of exceptional cytokine upregulation during the ribosomal dysfunction at various levels of gene regulation, including transcriptional signaling activation triggered by organellar distress, posttranscriptional upregulation, and favorable posttranslational processing of cytokines. Investigations of the molecular patterns of cytokine induction by ribosomal inactivation will improve our understanding of typical stress-induced processes of inflammatory signals and provide new insight into therapeutic targets for ribosome-related inflammatory diseases.

## 2. Organellar Sentinels for Cytokine Induction: Ribosome and ER

Some surface signaling receptors are gate keepers against intracellular stresses. Ribosomal inactivation by factors such as UV irradiation increases phosphorylation of the cytoplasmic membrane receptor, epidermal growth factor receptor (EGFR), on tyrosine residues to above basal levels, leading to activation of protein kinase B (PKB/Akt1) and downregulation of the Ras-extracellular signal-regulated kinase (ERK) signaling cascade [34]. Ribosome-inactivating palytoxin interacts with high affinity cell-surface receptors, including the Na^+^, K^+^-ATPase, or Na^+^/H^+^ antiporter [35–37]. These ribosomal inactivation-linked surface receptors (EGFR and Na^+^, K^+^-ATPase) can also trigger their downstream kinase signaling pathways, leading to proinflammatory cytokine production [38, 39]. Although surface receptors may be activated by ribosomal inactivation, most signaling sentinels that respond to ribosome-inactivating stressors are associated with organelles themselves, including the ribosome and endoplasmic reticulum (ER).

### 2.1. Ribosomal RNA Cleavage and PKR-Linked Sentinel for Ribosome-Inactivating Stress

Early responses to ribosome-inactivating stress activate the ribosome-based scaffold signaling protein network, resulting in diverse biological patterns including apoptosis and cytokine induction [40–42]. In addition to the modification or cleavage of ribosomal RNA, a variety of ribosome-inactivating stresses lead to phosphorylation of serine 51 on the alpha subunit of eukaryotic translation initiation factor 2 (eIF2), leading to global translational arrest [31]. The *α* subunit of eIF2 in the ribosome-based scaffold protein complex is the target of different stress-related mammalian protein kinases including double-stranded RNA-dependent protein kinase R (PKR) and protein kinase RNA-like endoplasmic reticulum kinase (PERK). Ribosome-inactivating stressors trigger an eIF2*α* kinase PKR which is recruited into ribosomal protein complex during cellular pathogenic stresses in response to the inflammatory stimulation [41, 43, 44]. PKR is an interferon-induced serine/threonine protein kinase activated by double-stranded RNA (dsRNA) [45] that plays important roles in the antiviral defense by interferon, particularly during cell growth control and differentiation [46, 47]. Mainly, dsRNA mediates PKR activation upon viral infection, which blocks the synthesis of new viral particle proteins [48]. Ribosome-inactivating stress is another inflammatory trigger known to activate PKR-linked signaling pathways in the ribosome [41, 49, 50]. Since activated PKR mediates proinflammatory chemokine induction in response to viral infection, it increases infiltration of inflammatory cells including neutrophils which promotes tissue injuries in response to viral infection [41, 51]. Proinflammatory chemokines such as MCP-1 and IL-8 induced by ribosomal inactivation thus exacerbated viral bronchopneumonia induced by respiratory reovirus infection [51]. Mechanistically, ribosomal inactivation damages the loops in the ribosome, which facilitates ribosomal binding to one or both dsRNA-binding domains of PKR and induces enzymatic activation [41]. While acute exposure to high levels of ribosomal stress, activated PKR plays important roles in activating stress responses like cell death via mitogen-activated protein kinases (MAPKs) such as p54, p46, and c-Jun N-terminal kinase 1 and 2 (JNK1/2) [50], milder exposure to ribosomal inactivation can trigger mucosal and systemic inflammation via the production of proinflammatory chemokines by epithelial and other immune-related cells [27, 29, 30, 52]. Low levels of ribosomal insults promote proinflammatory cytokine induction via a different set of MAPKs such as p38 [40, 41]. One upstream activator of p38 that responds to ribosomal stress is PKR, which is critical to ribosomal recruitment of p38, its subsequent phosphorylation, and p38-mediated transcriptional activation of proinflammatory cytokines [40]. In response to ribosomal inactivation by deoxynivalenol, ribosome recruits the hematopoietic cell kinase that also activates p38 MAP kinase cascade in macrophages [40]. Therefore, ribosomal 40S subunit serves as a scaffold for PKR and other recruited signaling molecules, facilitating MAPK mobilization and subsequent cytokine induction. However, more definite molecular mechanisms should be addressed to identify the link between ribosome-specific activation of PKR and ribosomal inactivation in future studies.

### 2.2. ER Stress-Related Sentineling Signals for Cytokine Induction by Ribosomal Inactivation

Ribosomes that synthesize proteins become bound to ER membrane, after which the two organelles engage in crosstalk related to various stress signals and the protein synthesis process [2, 3]. Activated ribosomal proteins thus may induce ER stress-related responses, which are attenuated by deletion of ribosomes in yeast and human cells such as monocyte-derived cells and epithelial cells [2, 3, 53]. ER-disrupting environmental and genetic factors cause accumulation of misfolded and unfolded proteins in the ER lumen, a condition termed ER stress. Ribosomal inactivation can also alter ER functions, and some chemical ribosomal inactivators such as trichothecenes, verotoxins, or ricin enhance unfolded protein responses that contribute to proinflammatory cytokine production and apoptosis-linked tissue injuries [54–56]. ER stress is positively associated with chronic proinflammatory diseases [57–59]. In particular, ER stress is a risk factor of inflammatory bowel diseases (IBDs) including Crohn's disease and UC, which are triggered by genetic or environmental factors such as smoking, stress, diet, and microbial components that can induce excessive inflammation [60–63]. Mechanistically, proinflammatory cytokines play central roles in mediating ER stress-linked inflammatory diseases [64–66]. Moreover, unlike canonical nuclear factor kappa B (NF-*κ*B) activation, the upstream activator I*κ*B*α* kinase (IKK) is not activated during ER stress [66]. Instead, the level of basal IKK activity maintained via an ER stress sensor inositol-requiring ER-to-nucleus signal kinase 1 (IRE1) is essential to regulation of NF-*κ*B activation. Phosphorylated eIF2*α* by PERK in combination with IRE1 action then leads to repressed synthesis of I*κ*B*α* and subsequent maximum NF-*κ*B activation during ER stress in monocyte-derived cells [66, 67]. Although macrophage NF-*κ*B is activated by ribosomal insults, epithelial NF-*κ*B expression and activity are strictly regulated to prevent overstimulation of proinflammatory responses following exposure to commensal bacteria [68, 69]. However, suppressed NF-*κ*B in gut epithelial cells is not beneficial during pathogen infection since the production of many antibacterial mediators such as defensins is dependent on NF-*κ*B signaling pathways for their induction in gut barrier. In spite of NF-*κ*B suppression some of epithelial proinflammatory chemokines such as IL-8 are upregulated by ribosomal inactivation [27, 70, 71]. This NF-*κ*B-independent cytokine induction will be further explained in the next section.

Moreover, ribosomal inactivation can trigger the expression of ER stress-linked transcriptional regulators such as CCAAT/enhancer-binding protein homologous protein (CHOP), which can mediate toxic inflammatory responses in human intestinal mucosa, lung, and pancreas [72, 73]. Although CHOP is a key apoptotic signal inducer, it can also modulate different types of inflammatory responses. Specifically, CHOP is a dominant negative form of C/EBP family members that lacks DNA binding activity and can form heterodimer complexes with other C/EBP members, thereby inhibiting their functions as transcription factors. Since C/EBP*β* mediates expression of anti-inflammatory peroxisome proliferator-activated receptor gamma (PPAR*γ*) by forming homodimers and binding to the PPAR*γ* promoter [74, 75], CHOP-C/EBP*β* complex thus interferes with basal PPAR*γ* expression and facilitates NF-*κ*B activation by ER stress [76]. Overall, ribosomal inactivation-induced ER stress enhances proinflammatory cytokine production via NF-*κ*B activation in monocyte-derived cells, which can be also facilitated by CHOP-mediated regulation of anti-inflammatory PPAR*γ*. By contrast, ribosomal inactivation may suppress epithelial NF-*κ*B expression and activity while some of epithelial proinflammatory chemokines are enhanced in NF-*κ*B-independent ways.

## 3. NF-***κ***B-Independent Transcriptional Regulation of Cytokine Induction

Once activated by early sentinels from ribosomal recruitment of signaling mediators or the provoked ER stress sentinels, the downstream MAPK cascade accelerates the induction of proinflammatory cytokines through activation of transcription factors such as NF-*κ*B, activating protein 1 (AP-1), CCAAT enhancer binding protein (C/EBP), cyclic AMP response element binding protein (CREB), and early growth response 1 (EGR-1) gene product [27, 33, 77–79]. Ribosomal inactivation activates NF-*κ*B-linked proinflammatory signals for cytokine induction in monocyte-derived cells, while extended exposure to the toxic stress can suppress the signals in epithelia as commented [80, 81]. Moreover, induction of epithelial proinflammatory cytokines by ribosomal inactivation occurs independently of the NF-*κ*B signaling pathway [27, 28]. Instead of NF-*κ*B, EGR-1 can be involved in proinflammatory chemokine gene expression in ribosomal inactivation-insulted intestinal epithelial cells. Ambivalent roles of epithelial EGR-1 were recently addressed in response to mucosal ribosomal stress [68]. While EGR-1 positively mediates epithelial chemokine induction by ribosomal inactivation, EGR-1 also contributes to negative regulation of proinflammatory NF-*κ*B signaling via PPAR*γ* induction in intestinal epithelial cells. EGR-1 is known to specifically bind and transactivate PPAR*γ* promoter [82, 83] although it can also be involved in inhibition of PPAR*γ* gene expression in some cell types [84]. Ribosomal inactivation disrupts the balance between PPAR*γ* and NF-*κ*B-linked signaling in the mucosal epithelia by enhancing EGR-1 gene expression and subsequently PPAR*γ* levels, leading to greater suppression of proinflammatory NF-*κ*B signaling in response to infectious agents including endotoxins [68]. Clinical investigations demonstrated that the commensal microflora can enhance the expression of PPAR*γ* that is impaired in ulcerative colitis (UC) patients, particularly in the enterocytes [85–87]. Since epithelial PPAR*γ* plays protective roles against the colonic inflammatory responses to both commensal and pathogenic bacteria [88–90], its attenuation by ribosomal insults would be detrimental as implicated in UC patients. Although PPAR*γ* generally attenuates epithelial inflammatory responses by triggering nuclear export of p65 protein in complex with PPAR*γ* [91], it can also regulate proinflammatory cytokine production via NF-*κ*B-independent activation of signaling mediators such as protein kinase C alpha, which induces cellular desensitization to proinflammatory stimulation in monocyte-derived cells [92]. Overall, ribosomal inactivation triggers chemokine gene induction through NF-*κ*B or alternate proinflammatory transcription factors including EGR-1, while negatively regulating PPAR*γ*-directed anti-inflammatory actions.

## 4. Posttranscriptional Regulation

In addition to transcriptional regulation, posttranscriptional modifications such as mRNA stabilization may lead to cytokine superinduction via ribosomal inactivation in leukocytes and gut epithelial cells [69, 93, 94]. Moreover, epithelial ER stress that can be triggered by ribosomal inactivation also enhances cytokine mRNA stability [95]. In response to ER stress, eukaryotic cells selectively shut down the global protein translation via eIF2*α* phosphorylation, which results in a limited availability of the eIF2-GTP-tRNA^Met^ complex [96]. However, mRNA of some early stress responsive genes is rapidly recruited from translating ribosomes into stress granules (SGs) as untranslated form [95, 97]. Independently of eIF2*α* phosphorylation, blocking of ribosome recruitment also induces stress granule formation by interfering with eIF4B activity, an RNA helicase required for the ribosome recruitment phase of translation initiation [98, 99]. Therefore, disruption of ribosomal integrity as an alternate pathway would induce the formation of SGs without regard to stress-induced eIF2*α* phosphorylation. The temporal storage in stress granules provides mRNA with shelter from degradation and maintain silenced mRNAs to resume protein translation upon stress release. The untranslated mRNAs in SGs under stress are protected via SG-recruited mRNA-stabilizing proteins such as HuR/Elav-like RNA binding protein 1 (ELAVL1) that positively regulates the stability of mRNA transcripts containing AU-rich elements (AREs), including those for proinflammatory cytokines [100–102]. HuR protein binds to AREs in the 3′ untranslated region (3′UTR) of target mRNA molecules in the nucleus and then translocates into the cytoplasm for translation. Cytosolic translocation of the HuR protein is also initiated by mucosal ribosome-inactivating stress and ultimately stabilizes cytokine mRNA in the cytoplasm and SGs [71, 93]. As an HuR modulator, CHOP plays key roles in maintenance of the mRNA stability of cytokine genes in response to ribosome-inactivating stress [71]. Similar to ER stress, ribosome-inactivating stress induces CHOP expression, which also suppresses PPAR*γ* expression as indicated in Section 2. As a target of CHOP induced by ribosomal inactivation, PPAR*γ* is not involved in epithelial chemokine regulation at the transcriptional level. Instead, CHOP is a positive regulator of cytokine mRNA transcript stability that occurs via HuR protein [71, 76]. In response to mucosal ribosomal inactivation, enhanced mucosal PPAR*γ* regulates epithelial chemokine gene induction, which is posttranscriptionally modulated by HuR independently of NF-*κ*B-linked signals [71, 103]. Mechanistically, induction of CHOP by ribosomal inactivation enhances the cytosolic translocation of HuR protein by suppressing PPAR*γ* expression. Because PPAR*γ* inhibits the cytosolic translocation of HuR, suppression of PPAR*γ* by CHOP protein facilitates HuR movement into the cytoplasm and subsequent cytokine mRNA stabilization [71]. Ribosome inactivation-triggered translocation of HuR protein also enhances expression of activating transcription factor 3 (ATF3), which plays a central role in the regulation of proinflammatory NF-*κ*B signals in human gut epithelial cells [102]. Therefore, HuR contributes to cytokine superinduction by ribosome-inactivating stress while retarding NF-*κ*B activation via ATF3. Further studies are needed to assess the effects of altered PPAR*γ* on the stability of chemokine transcripts in addition to the regulatory action of PPAR*γ* on transcriptional activity of chemokine genes in response to mucosal ribosomal inactivation. Taken together, ribosomal inactivation leads to chemokine superinduction via mRNA stabilization by RNA-binding proteins such as HuR whose cytosolic translocation is facilitated by CHOP protein. Moreover, HuR can mediate ATF3 superinduction as well, leading to suppression of NF-*κ*B signals in enterocytes.

## 5. Posttranslational Processing of Proinflammatory Cytokines and Cytokine Receptors

### 5.1. Roles of Inflammasomes in Cytokine Processing

Following transcriptional induction, some proinflammatory cytokines such as interleukin 1*β* (IL-1*β*) and interleukin 18 (IL-18) undergo maturation triggered by the inflammasome-linked sentinel [104–106]. Ribosome-inactivating stress also triggers some inflammasome-linked processes that promote the maturation of inflammatory cytokines in monocyte-derived cells [107–109]. Ricin, a potent ribosomal toxin, leads to acute lung injury and symptoms resembling acute respiratory distress syndrome via the IL-1*β*-activated signaling pathway in alveolar macrophages [110]. Mechanistically, the nod-like receptor (NLR) family member, NLRP3, stimulates IL-1*β* processing via NLRP3 inflammasome, which is triggered by ribosome-inactivating stress in pulmonary macrophages [107, 108]. Although the detailed molecular modes are still unknown, ribosomal inactivation may mediate a drop in cellular potassium, leading to protein translation and subsequent activation of the NLRP3 inflammasome [108]. A recent study suggested that, as another mechanism of inflammasome activation by ribosomal inactivation, the activation of p38 MAPK by ribosome-inactivating stress triggers formation of a pyrin inflammasome complex with ASC: apoptosis-associated speck-like protein containing a caspase recruitment domain and procaspase-1, leading to ASC oligomerization, caspase-1 activation, and pro-IL-1*β* processing in macrophages [109]. In both cases, ribosomal inactivation is considered to provoke latent inflammatory storms via inflammasome activation in the monocyte-derived cells.

### 5.2. Ribosomal Inactivation-Triggered Ectodomain Shedding of Cytokine Receptor

Ribosomal inactivation stimulates MAP kinase signaling via direct modulation of a broad spectrum of physiological stimuli including appropriate growth factors and cytokines [10, 32, 111]. Activated MAP kinase triggers phosphorylation of TNF-*α*-converting enzyme (TACE), which is also known as a disintegrin and cell-surface metalloproteinase 17 (ADAM17), which is then translocated to the cell surface for its action. TACE-dependent ectodomain shedding of cell-surface proteins is increased by ERK and p38 MAP kinase, which phosphorylate threonine 735 in the cytoplasmic tail of TACE [112, 113]. Ligands of the epidermal growth factor receptor (EGF) need to be cleaved by TACE and shed as soluble proteins in order to become systemically available and many proinflammatory cytokines or receptors of the tumor necrosis factor alpha (TNF*α*) family are also cleaved by TACE [113–115]. Consistent with these findings, ribosomal inactivation activates TACE-mediated ectodomain shedding of TNF receptor 1 via activated MAP kinases in pneumocytes [116, 117]. In this case, ribosomal inactivation attenuated cellular responses to proinflammatory cytokines via TACE-mediated shedding of cytokine receptor, which regulates the storm of proinflammatory cytokines in the body that occurs under the ribosomal-inactivating stress. However, TACE is also needed for the generation of soluble TNF*α*, an important therapeutic target of inflammatory diseases such as arthritis, sepsis and colitis and thus the inhibition of TACE leads to protection from the diseases in animals [118] although many physiologic activities of TACE make blockade of this enzyme problematic [119]. Since TACE-associated cytokine regulation could be thus beneficial or detrimental, more careful investigations are needed for the ultimate actions of the ribosomal inactivation in the TACE-related pathogenesis.

## 6. Conclusion

**Figure 1:**
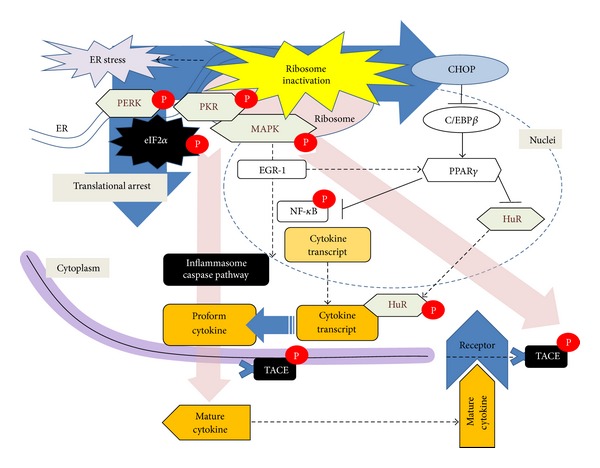
A putative diagram of ribosomal stress-derived signaling networks for cytokine induction.

Although the primary effects of ribosomal inactivation on cells are linked to global protein synthesis, some early responsive gene products including proinflammatory cytokines are exclusively enhanced in lymphoid tissue and epithelia. Although surface receptors triggering cytokine induction are activated by some ribosomal inactivation, most primary responses originate from the ribosome and ER (Figure 1). The ribosomal subunit serves as a scaffold for PKR, while other signaling mediators help promote their activation, which then facilitates MAPK mobilization and subsequent cytokine induction. Ribosomal inactivation-induced ER stress also can mediate proinflammatory cytokine production via NF-*κ*B activation, which is facilitated by CHOP-mediated regulation of anti-inflammatory PPAR*γ*. In addition to NF-*κ*B-linked signals, cytokine expressions are transcriptionally promoted by alternate proinflammatory transcription factors such as EGR-1 but paradoxically EGR-1 enhances PPAR*γ* expression, which suppresses proinflammatory NF-*κ*B signaling in gut epithelial cells. In addition to transcriptional regulation, posttranscriptional modifications such as mRNA stabilization contribute to cytokine superinduction via various RNA-binding stabilizers such as HuR for stabilization of mRNA transcripts with cytokine transcript AREs. Moreover, cytokine mRNA stability is enhanced by CHOP protein, which facilitates cytosolic translocation of HuR by limiting PPAR*γ* expression in cells under ribosome-inactivating stress. Cytokine protein processing is another critical regulation by ribosomal insult. Some proforms of cytokines such as IL-1*β* and IL-18 are cleaved to mature active forms by inflammasome-triggered caspases, which are also activated by ribosomal inactivation in macrophages. However, since ribosomal inactivation causes shedding of the ectodomain of several cytokine receptors via MAPK-activated cell-surface metalloproteinase TACE, additional information is needed to understand whether the ribosomal inactivation would attenuate or exacerbate the stress-associated inflammatory diseases.
